# Smokeless tobacco industry's brand stretching through FM radio: A study from Delhi National Capital Region, India

**DOI:** 10.3389/fpubh.2022.999552

**Published:** 2022-10-14

**Authors:** Prashant Kumar Singh, Rupal Jain, Akansha Tyagi, Amit Yadav, Shalini Singh

**Affiliations:** ^1^Division of Preventive Oncology and Population Health, WHO FCTC Knowledge Hub on Smokeless Tobacco, ICMR-National Institute of Cancer Prevention and Research, Noida, Uttar Pradesh, India; ^2^Guru Jambheshwar University of Science and Technology, Hisar, India; ^3^The International Union Against Tuberculosis and Lung Disease (The Union) South East Asia Office, New Delhi, India

**Keywords:** brand stretching, surrogate advertisement, smokeless tobacco (SLT), tobacco industry, radio station, India

## Abstract

Smokeless tobacco (SLT) consumption is associated with multiple adverse health effects and mortality, with the highest burden in India. The WHO FCTC has banned tobacco advertisement, promotion and sponsorship *via* Article 13. Indian laws also prohibit any kind of direct or indirect advertisements in all forms of audio, visual, and print media; brand promotion; and sponsorship of tobacco products. However, the tobacco industry continues to find aggressive marketing ways to advertise their products. This study aims to assess the extent of surrogate advertisement of smokeless tobacco products through frequency modulation (FM) radio stations in the city of Delhi (National Capital Territory of India). In this study, the advertisements broadcasted over FM radio across different channels (private and government owned) in total 162 h were analyzed. The time duration was spread evenly over morning peak hours, off hours, and evening peak hours. It was found that multiple brands including Vimal, Kamla Pasand, and Rajshree have used surrogate advertising to market their brands that are commonly associated with smokeless tobacco products. However, no such advertisement was found to be aired on government-owned FM channels. The total surrogate advertisements broadcasted were over 286 times, where no significant difference was found in distribution among peak and non-peak hours. The study indicated that the tobacco industry is making its way to sell the products through indirect advertisements. The need of the hour is to not only enforce tobacco advertising ban laws in all forms of advertising media but also to strictly regulate smokeless tobacco products.

## Introduction

Smokeless tobacco (SLT) are non-combustible tobacco products, which are highly addictive in nature and can be consumed orally or nasally (1). SLT is consumed by 365 million people across the globe and has caused 0.35 million deaths in the year 2017 (2). The consumption of SLT is widespread; however, low- and middle-income countries (LMICs) share the largest burden, with nearly 82% users in southeast Asia (3). With the presence of more than 30 carcinogens in high concentrations in SLT products, it can cause adverse health outcomes, including cancer of oral cavity, pharynx, esophagus, and pancreas (1), with increased risk of oral health problems like leukoplakia, gingivitis, periodontitis, and dental caries (4). About 50% of oral cancer cases in India are traceable to SLT consumption (5). Increased risk of cardiovascular deaths, stillbirths (6), and other negative reproductive outcomes are also found to be associated with the consumption of SLT (4). Due to such detrimental effects, the World Health Organization Framework Convention for Tobacco Control (WHO-FCTC) has recommended to ban tobacco advertisement, promotion, and sponsorship under Article 13 in order to reduce the tobacco consumption (7).

According to the Global Adult Tobacco Survey (GATS) 2016–17, India, with around 200 million consumers, is one of leading SLT producers and consumers, representing about two-thirds of the global consumption of SLT (8). Across the globe, 70% of the total disease burden caused by SLT in terms of disability-adjusted life years (DALY) loss is borne by India (2). The government of India implemented Cigarettes and other Tobacco Products Act 2003 (COTPA) Section 5, which prohibits any kind of direct or indirect advertisement in all forms of audio, visual, and print media; brand promotion; and sponsorship of tobacco products (9) and also ratified the WHO FCTC in the year 2004. Yet, the tobacco industry has found creative ways to aggressively advertise and market their products, especially through brand stretching.

Brand stretching is a technique when a company extends one of its established brand names or identity to another product category. When such a strategy is applied to tobacco products, it can also be referred to as surrogate advertisement, which literally means “substitute.” Surrogate advertisement of tobacco products includes using an established brand name or identity associated with any tobacco product for other non-tobacco products (e.g., pan masala, elaichi (cardamom seeds), areca nut, dates, and mouth fresheners). In this article, the terms, “brand stretching” and “surrogate advertisement” are used interchangeably due to their similar kind of interpretation in regard to tobacco products.

There are studies that highlight different marketing strategies adopted by tobacco companies in anticipation of the implementation of COTPA in India, their prospective target population, and their influence on them (10–13). One of them emphasized the usage of similar color schemes for tobacco and non-tobacco products, and sponsoring events as a means of marketing (12). But these studies only focused on print and audio-visual media, despite radio being less expensive and having greater reach as a medium of communication and entertainment in LMICs than television (14). To address this gap, the present study aims to assess the extent of surrogate advertisement of SLT products, specifically through audio media, which is broadcasted using popular frequency modulation (FM) radio stations in the city of Delhi (National Capital Territory of India).

## Methodology

The study utilized the advertisements broadcasted over FM radio across different channels, selected on the basis of ownership (government/private) and popularity/rating. The nine channels included in the study with their frequencies are Radio City (91.1), Hit FM (95), Radio Nasha (107.2), Radio Mirchi (98.3), Fever 104 FM (104), AIR FM Gold (106.4), Red FM (93.5), Radio One (94.3), and AIR FM Rainbow/Akashvani (102.6).

The data collection took place from 7 July 2021 to 20 August 2021. Each channel was surveyed for 2 days; thus, took 18 days to cover all the 9 channels. These channels were listened to for 9 h every day, and instances of surrogate advertisements aired over these channels in a total of 162 h were observed for analysis. The time durations for documentation were spread evenly over morning peak hours (8 am to 11 am), afternoon hours (12 pm to 3 pm), and evening peak hours (5 pm to 8 pm). Information about time and frequency of the advertisement, details of the advertisement (brand name/product endorsed) and the radio station were documented, and analysis was conducted using Microsoft Excel.

### Ethical consideration

The present study does not require an ethical review as there were no human beings or animals involved as participants in the study. The researchers have listened to publicly available data aired on different FM radio channels and documented the same in due course for analysis.

## Results

It was found that multiple companies have used the technique of brand stretching to market their tobacco products *via* FM radio. The total occurrence of surrogate advertisements broadcasting was 286 times, but no such instance was recorded to be aired on government-owned FM channels (Table 1). Thus, the average frequency of surrogate advertisement per day during study observation (excluding government-owned channels) was estimated to be 20.42. There was no significant difference found in overall distribution among peak and non-peak hours. However, 10 am to 11 am was found to be the busiest hour for surrogate advertisements with maximum frequency (*n* = 44), and 8 am to 9 am was found to have the lowest frequency (*n* = 22).

**Table 1:** Frequency of the surrogate advertisement broadcasted across different FM Radio channels selected in the present study.

**Radio Channel**	**Day-1**				**Day-2**				**Frequency per hour**
	**Morning hours**	**Afternoon hours**	**Evening hours**	**Total frequency**	**Morning hours**	**Afternoon hours**	**Evening hours**	**Total frequency**	
Radio City	4	7	7	18	9	8	6	23	2.28
HIT FM	5	5	5	15	13	10	10	33	2.67
Radio *Nasha*	7	8	8	23	4	5	8	17	2.22
Radio *Mirchi*	7	9	6	22	5	7	3	15	2.06
Fever 104 FM	3	3	5	11	4	3	4	11	1.22
Red FM	7	9	7	23	13	14	14	41	3.56
Radio One	7	8	5	20	6	4	4	14	1.89
AIR FM GOLD (government owned)	0	0	0	0	0	0	0	0	0
AIR FM Rainbow (government owned)	0	0	0	0	0	0	0	0	0

The brands which used such strategies included Vimal with the maximum number of frequency (82%), followed by Kamla Pasand (10%) and Rajshree (8%), where Vimal covered over 235 instances of advertisements out of 286 (Table 2). Elaichi (cardamom seeds) as mouth freshener was the most endorsed product (89.5%), while remaining advertisements included brand name, tagline, or any other surrogate product (10.5%). In a few cases, companies also used the means of sponsoring other shows on FM radio in pursuance of increasing brand visibility. However, it is interesting to note that a big share of mouth freshener promotions targeted specifically women either by using female voices or by advertising women-specific products.

**Table 2:** List of the companies documented in the study utilizing the strategy of surrogate advertisement.

**Brand**	**Type of advertisement**		**Frequency**	**Percentage**
	**Brand name**	**Product endorsed**		
Vimal	2	233	235	82%
Kamla Pasand	28	0	28	10%
Rajshree	0	23	23	8%

## Discussion

Advertising is a vital marketing technique, which has been used for centuries to influence people's emotions and consumption behavior (15, 16). Industries use different tactics of entertainment, familiarity, social imaging, and advertisement spending to have a direct impact on consumer buying behavior (17). Similarly, exposure to tobacco advertisement is found to be associated with initiation as well as consumption of tobacco. This association is even more significant for young adults from vulnerable group (i.e., living below the poverty level and lower education level) (18). This evidence is also supported by our study where we found voice-overs of Bollywood actors have been used to allure the sentiments of particularly young audience. Phrases targeting the social desirability among youth like “*sab banenge follower”* (be the trend setter) and “*tashan ka jashan”* (celebrate your attitude), or relating to unity like “*bhashayein rang roop hai juda, par humari zubaan hai ek”* (diverse by origin and language, but united by taste) were used frequently in the advertisements. Studies also suggest that women are more likely to get influenced by advertisement for consumption of smokeless tobacco than men (19). This could be one of the reasons that a considerable share of the products documented in this article are found to be targeting women explicitly. More specifically, our findings indicate that more than half of the voice-overs used in the tobacco advertisements were of women, reiterating that the industry is putting their best efforts to lure women.

In order to combat the increase in tobacco consumption in India, tobacco advertisement has been banned under COTPA, Section 5, which is in line with the Article 13 of the FCTC and the guidelines therein. Tobacco companies utilize different channels of communication to persuade the consumers including print, audio, and audiovisual media. Studies suggest that familiarity to the brand name can have a significant psychological impact on the consumer, leading to positive buying behavior (17). Considering this, in 1995, the government of India had enacted the Cable Television Networks (Regulation) Act to regulate direct or indirect promotion of tobacco products on cable television networks. However, the regulation was relaxed for “genuine brand extensions” in 2009 and is in conflict with COTPA 2003 and its regulations besides being in violation of the WHO FCTC obligations. Unfortunately, marketing techniques involving using the same brand name and color composition still continue to be used by the tobacco companies, which are referred to as brand stretching.

In this study, elaichi (cardamom) as mouth freshener is the most endorsed product over various FM radio stations through brand extension of products. As observed in the Indian context, mouth fresheners (elaichi) are used after and in between meals as a traditional practice and are socially acceptable (20). This could be one of the reasons that the current study observed that elaichi was utilized more for promotion than any other surrogate products like pan masala or areca nut. During this study, it has also been observed that the tobacco companies have targeted to increase their familiarity and brand visibility among the population by means of sponsoring different shows broadcasted, despite its prohibition *via* the WHO FCTC Article 13 (7). Regardless of multiple laws altogether putting a comprehensive ban on tobacco advertisement, promotion, and sponsorship in order to safeguard the public from the tobacco epidemic, India could not succeed entirely. A research based in Europe indicates that stringent ban on tobacco advertisement along with regulation of supply, distribution, and smoke-free laws has led to steep decline in consumption of tobacco (21).

Despite the presence of laws in India for banning tobacco advertisement, promotion, and sponsorship, copious violations have been found frequently during this study. Thus, there is a need for a clear and uniform policy integrating different laws and regulations necessary to reduce tobacco advertising and consumption. However, in this study, no instance of brand stretching or tobacco advertisement on government-owned FM channels was found. This throws incandescent attention to the existing loopholes or breach of tobacco control laws in the private sector. Thus, it is salient to not only enforce the laws related to the ban on tobacco advertising but also regulate them strictly on private platforms. Furthermore, protocols to penalize direct or indirect violators of such laws should be made more stringent to ensure greater compliance with the legal provisions.

## Limitations

The present study is based on the data documented for 9 h per day bifurcating into 3 h each for morning peak hours, afternoon hours, and evening peak hours. These 9 h assumed to be a representation of an entire day and can be extrapolated to 24 h; however, this assumption can also be counted as a study limitation. Apart from this, the study is conducted using radio channels based in the National Capital Territory of India, Delhi, where there is a possibility of high surveillance in comparison to other geographical locations, including small towns of India. Thus, there is a need to conduct this study on a large scale at different geographical locations in order to incorporate the skewness in distribution.

## Data availability statement

The original contributions presented in the study are included in the article/supplementary material, further inquiries can be directed to the corresponding author.

## Author contributions

All authors listed have made a substantial, direct, and intellectual contribution to the work and approved it for publication.

## Conflict of interest

The authors declare that the research was conducted in the absence of any commercial or financial relationships that could be construed as a potential conflict of interest.

## Publisher's note

All claims expressed in this article are solely those of the authors and do not necessarily represent those of their affiliated organizations, or those of the publisher, the editors and the reviewers. Any product that may be evaluated in this article, or claim that may be made by its manufacturer, is not guaranteed or endorsed by the publisher.
